# Shear-Induced Cycloreversion Leading to Shear-Thinning and Autonomous Self-Healing in an Injectable, Shape-Holding Collagen Hydrogel

**DOI:** 10.1021/acsami.4c08066

**Published:** 2024-10-08

**Authors:** Mahsa Jamadi Khiabani, Sareh Soroushzadeh, Ardeshir Talebi, Ayan Samanta

**Affiliations:** 1 Macromolecular Chemistry, Department of Chemistry—Ångström Laboratory, Uppsala University, Box 538, 751 21 Uppsala, Sweden; 2 Department of Pathology, School of Medicine, Isfahan University of Medical Sciences, Isfahan 8174673461, Iran

**Keywords:** click chemistry, bio-orthogonal reaction, collagen hydrogel, furan−maleimide Diels−Alder reaction, shear-thinning, autonomous self-healing, cardiac tissue engineering, cycloreversion, mechanophore, shape-holding, injectable

## Abstract

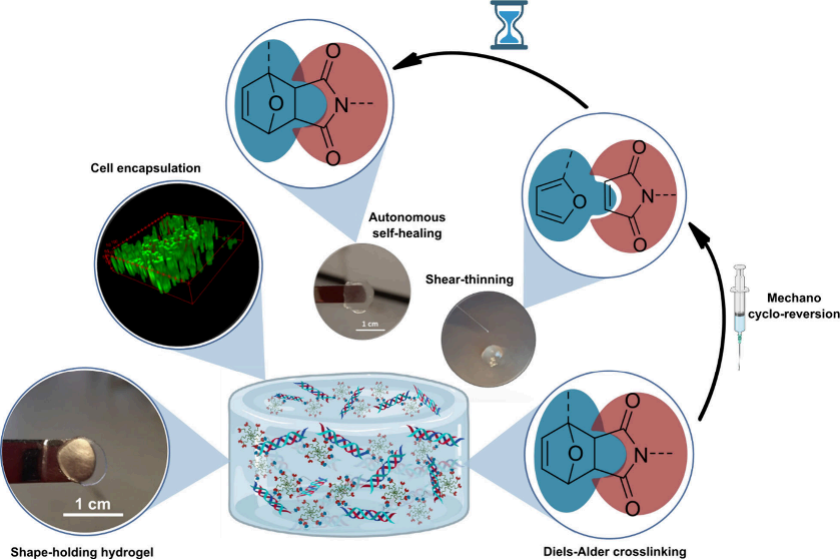

*In vivo* injectable extracellular matrix (ECM) derived hydrogels that are suitable for cell encapsulation have always been the holy grail in tissue engineering. Nevertheless, these hydrogels still fall short today of meeting three crucial criteria: (a) flexibility on the injectability time window, (b) autonomous self-healing of the injected hydrogel, and (c) shape-retention under aqueous conditions. Here we report the development of a collagen-based injectable hydrogel, cross-linked by cycloaddition reaction between furan and maleimide groups, that (a) is injectable up to 48 h after preparation, (b) can undergo complete autonomous self-healing after injection, (c) can retain its shape and size over several years when stored in the buffer, (d) can be degraded within hours when treated with collagenase, (e) is biocompatible as demonstrated by *in vitro* cell-culture, and (f) is completely resorbable *in vivo* when implanted subcutaneously in rats without causing any inflammation.

## 1 Introduction

In recent years injectable hydrogels have gained enormous interest in the field of biomaterials as they can be administered through a minimally invasive surgery and can fill in defects of any size and shape.^1^ However, *in vivo,* injectability through a 27-gauge needle and quick setting of the hydrogel at the injection site would require the hydrogel precursor solutions to have low enough viscosity to allow passing through the narrow needle smoothly and yet form a hydrogel almost immediately to prevent dissolution of the hydrogel and burst release of the encapsulated cells or drugs. Moreover, the injected hydrogel should be degradable in the host to avoid causing foreign body response which can lead to fibrosis.^2^ Additionally, if the injectable hydrogel is to be used for cell delivery, the gelation should be slow enough to allow proper mixing and even distribution of cells within the matrix but fast enough to prevent agglomeration and sedimentation of the cells.^3^ Extensive efforts are thus often dedicated to fine-tuning the gelation time by varying the number of cross-linkable groups in the polymer chain, altering total polymer concentration in the hydrogel, varying catalyst concentration, or using external or *in vivo* stimuli.^4−7^ Moreover, the hydrogel should be shear-thinning to protect the encapsulated cells from the shear stress exerted during the injection and autonomously self-healing to restore the hydrogel mechanical properties, resulting in a cohesive uniform hydrogel after injection. Encouraged by this idea, several shear-thinning and self-healing hydrogels were developed.^8^ Despite substantial developments, injectable hydrogels suffer from a major drawback; they swell and cannot hold their shape.^9^ Unwanted swelling exerts pressure on the surrounding tissues, resulting in edema-like conditions. Moreover, in most cases, the self-healing of the injected hydrogel requires an external trigger. Hence, there is an unmet need for injectable, shear-thinning, and autonomous self-healing hydrogels that hold their shape, do not swell, and are degradable in the host.

Hydrogels made from collagen, the most abundant component in the extracellular matrix (ECM), are demonstrated to promote the regeneration of various tissues and can be easily remodeled *in vivo*. Moreover, collagen bears focal-adhesion sites for cells, and therefore, collagen hydrogels do not require additional functionalization with bioactive peptides for cell spreading and proliferation.^10^ While collagen can self-assemble, a process known as fibrillation, at physiological pH, temperature, and salt concentration to form a hydrogel, such physically assembled collagen hydrogels are too soft for surgical manipulations and result in excessive swelling and too quick dissolution and are prone to rapid enzymatic degradation.^11^ One potential solution to this problem would be to chemically cross-link the collagen molecules to form a hydrogel since permanent covalent bonds are more stable than physical interactions and should prevent excessive swelling and too rapid dissolution *in vivo*. Hence, we and others have developed chemically cross-linked collagen hydrogels employing the pendant amines and carboxylic acids from the side chains of collagen.^12−18^ However, such cross-linking chemistries developed to date are toxic. If such hydrogels need to be injected *in vivo*, the cross-linking reaction needs to be quenched before injection.^19,20^ This defeats the purpose of chemical cross-linking as often incomplete cross-linking leads to a very soft hydrogel. Alternatively, injection of the hydrogel after complete cross-linking results in a noncohesive crushed gel. In either case, cell-delivery using such hydrogels is highly challenging due to the toxic nature of the cross-linking chemistry.

Therefore, researchers have adopted a strategy where the collagen is modified with functional groups other than amines or carboxylic acids to be able to use less or nontoxic cross-linking reactions. A popular strategy relies on photoinitiated radical polymerization. Consequently, several modified collagen hydrogel systems were developed that require UV or visible light for radical-mediated cross-linking.^21−25^ However, the limited penetration depth of UV or visible light and generation of free radicals are noticeable drawbacks of these hydrogels.^26^ As a result, alternative systems that do not involve free radicals and rely on light will be valuable.

Recently, we have developed bio-orthogonally cross-linked injectable, shear-thinning, and shape-holding collagen hydrogels employing Michael addition reactions involving thiols and Michael acceptors such as maleimide or (meth)acryloyl functional groups.^27−29^ Some of them are partly self-healing due to the slow cross-linking and reversibility of the reaction in the presence of excess thiols.^27,29^ Nonetheless, a major drawback of hydrogel systems relying on thiols is the lack of control over side reactions involving thiols, especially oxidation of thiols to disulfides, which renders the storage of thiol collagen challenging. Furthermore, a complete and autonomous self-healing of the hydrogels after injection was not achieved.

The most promising approach to address the instability issue of thiol-collagen is to introduce collagen modifications that will undergo bio-orthogonal cycloaddition reactions without requiring light since the majority of the dienes and dienophiles are more stable than thiols. Furthermore, fast reaction rates and the absence of byproduct formation classify them as “click” reactions. In the past years, several examples include the use of cycloaddition reactions for the preparation of bio- and nanomaterials.^30^ Strain-promoted azide–alkyne cycloaddition (SPAAC) is of particular interest as injectable collagen hydrogel cross-linked using SPAAC has been developed for *in situ* filling of corneal defects and demonstrated to support the regeneration of corneal epithelium *in vivo*.^31,32^ However, these hydrogels are injectable only during the early phase of gelation and are not self-healing. This is attributed to the irreversible nature of SPAAC. Among other cycloadditions, the reaction between norbornene and tetrazine has been employed to prepare hydrogels from ECM-derived polymers.^33,34^ Similarly, these hydrogels were not injectable owing to the very fast and irreversible nature of the cross-linking reaction.

A notable example among cycloadditions is the reaction between furan and maleimide. Furan-maleimide cycloaddition reaction has been successfully employed earlier to develop cell-laden hydrogels,^35−39^ hydrogels emulating the ECM,^40^ tough hydrogels,^41^ and stimulus-dependent self-healing hydrogels^42^ and for sustained delivery of drugs or therapeutic factors.^43,44^ Furthermore, furan maleimide cycloaddition reaction has been recently used for preparing gelatin-based hydrogels^45,46^ and drug delivery^47^ and for developing nanoparticle-carrying hydrogels.^48−51^ However, a temperature as high as 65 °C was needed to perform the cross-linking leading to hydrogel formation. Furthermore, these hydrogels were noninjectable and lacked self-healing properties at ambient temperature and in some cases required incubation at 37 °C for 5 h to achieve self-healing following a single incision.^42^

To date, no example of an injectable collagen hydrogel exists that could undergo autonomous self-healing after injection. Although we have developed several bio-orthogonally cross-linked injectable, shear-thinning collagen hydrogels with shape-retaining properties, autonomous self-healing could not be achieved. Here we report an injectable collagen hydrogel cross-linked through the cycloaddition reaction between furan and maleimide which undergoes complete autonomous self-healing at ambient temperature after injection. The developed hydrogel is shear-thinning and can be injected in a gel state up to after 2 days of preparation. Furthermore, this hydrogel can hold its shape after prolonged storage in buffer and yet can be degraded within hours *in vitro* by collagenase and can be resorbed completely within two months *in vivo*. Last but not least, cardiac stem cells and endothelial cells can be encapsulated in these hydrogels without any noticeable toxicity.

## 2 Experimental Section

### 2.1 Materials

Furfuryl glycidyl ether (FGE), 2,4,6-trinitrobenzenesulfonic acid (TNBS), phosphate-buffered saline (PBS) powder, Trizma base powder, and collagenase from *Clostridium histolyticum* were purchased from Merck (Sweden). 8-arm PEG-maleimide (10 kDa) was purchased from Creative PEGWorks (USA). Porcine type I collagen (NMP collagen PS) was purchased from Nordic Biolabs (Sweden). The human umbilical vein endothelial cell line (HUVEC) was purchased from Thermo Fischer (Sweden). Human fetal cardiac mesenchymal stromal cells (hfcMSC) were obtained from Prof. Karl-Henrik Grinnemo. LIVE/DEAD viability/cytotoxicity kit and Alexa Fluor 594 Phalloidin were purchased from Invitrogen, Thermo Fisher (Sweden). 4,6-Diamidino-2-phenylindole (DAPI) was purchased from Merck (Sweden).

### 2.2 Synthesis and Characterization of Collagen Furan

Porcine type I collagen (molecular weight 300 kDa)^29^ was dissolved in water (0.5% w/v) and deoxygenated by bubbling argon through the solution. The pH was adjusted to 10 using NaOH (2 M). After cooling the reaction mixture in an ice bath, furfuryl glycidyl ether (FGE) (3 mol equiv with respect to lysine and arginine amines considering 114 amines from lysine and arginine combined per collagen molecule) was added dropwise while stirring and maintaining the pH at 10 by intermittent addition of NaOH (2 M). The reaction mixture was stirred at room temperature overnight in the dark under an argon atmosphere. The reaction mixture was then purified by dialysis using 12–14 kDa cutoff dialysis tubes (Spectrum Laboratories, Inc., CA, USA) against deionized water with pH 10 for 2 days and then against deionized water with pH 4.5 for another 2 days. The purified collagen furan solution was lyophilized to obtain an off-white fluffy solid, which was kept at −20 °C under argon until further use.

### 2.3 Nuclear Magnetic Resonance (NMR) and Fourier Transform Infrared (FT-IR) Spectroscopy

The ^1^H NMR spectroscopy was used to confirm the functionalization of collagen with FGE. Proton NMR spectra of collagen furan solution in D_2_O (1% (w/v)) were recorded using a JEOL JNM-ECP series FT NMR spectrometer at a magnetic field strength of 9.4 T, operating at 400 MHz for ^1^H NMR. Experimental conditions were as follows: 400 MHz, number of scans 20 000, delay 600 s, and the spectrum was recorded at 45 °C to reduce line broadening. The FTIR spectra of collagen and collagen furan were acquired using a Shimadzu IRTracer-100 in the range 400–4000 cm^–1^. Each spectrum was acquired with a resolution of 4 cm^–1^.

### 2.4 Trinitrobenzenesulfonic Acid (TNBS) Assay

The degree of modification of collagen furan was quantified using TNBS colorimetric assay as described earlier with minor adjustments.^28^ Briefly, 2 mg of dry sample was dissolved in 200 μL of deionized water, followed by mixing with 1 mL of NaHCO_3_ (4% w/w) and 1 mL of TNBS solution (0.5% w/w in water) at 40 °C under mild shaking and dark conditions for 4 h. Afterward, 3 mL of HCl (6 M) solution was added to quench the reaction, followed by three times extraction of unreacted TNBS from the sample with diethyl ether. UV absorbance of samples was recorded at 346 nm using a Lambda 35 UV/vis spectrophotometer (PerkinElmer, Sweden) against a blank prepared by the above procedure except that the HCl solution was added before the addition of TNBS solution. The same procedure was used for pristine collagen samples which were used as controls. The degree of functionalization was calculated as follows:
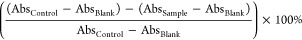


### 2.5 Circular Dichroism

Collagen furan and pristine collagen samples were dissolved in deionized water at a concentration of 0.02% (w/v) and monitored using a quartz cell of 0.1 cm path length in a JASCO J-1500 spectrometer (JASCO Corporation, Tokyo, Japan). Spectra were measured from 190 to 260 nm at 25 °C with a scan rate of 1 nm/s. The samples were corrected against a baseline obtained by measuring the deionized water.

### 2.6 Viscoelastic Behavior of Collagen Furan Solution

Effects of temperature on the viscoelastic behaviors of 2% (w/v) collagen furan solution were studied by dynamic oscillatory temperature sweeps using a Discovery Hybrid Rheometer 2 (TA Instruments, Sollentuna, Sweden). A 20 mm stainless steel parallel plate geometry was used. The dynamic oscillatory temperature sweeps were conducted at 1% strain and 1 Hz oscillation frequency, and the temperature was varied from 25 to 50 °C at a heating rate of 0.5 °C/min. The complex viscosity and the loss tangent were plotted against the temperature.

### 2.7 Preparation and Characterization of Hydrogel

Stock solution (10% w/w) of collagen furan was prepared in deionized water and stored at 4 °C. Hydrogels were prepared by mixing collagen furan stock solution and 8-arm PEG maleimide (10 kDa) in PBS using a syringe mixing system as described previously.^29^ The final concentration of collagen furan in the hydrogel was maintained at 2% (w/w). Four different formulations were prepared by changing the concentration of PEG maleimide in hydrogel to obtain a furan to maleimide ratio of 1:1, 1:2, 1:3, and 1:4 (**r1**, **r2**, **r3**, and **r4**). Collagen-furan gel was prepared by extruding ∼100 μL of the mixed components between two glass slides separated by a 1 mm spacer and cured overnight in a humid atmosphere and then stored in PBS at room temperature for 3 days before subjecting to further analyses.

### 2.8 Viscoelastic behavior of different hydrogel formulations

To study the viscoelastic properties of different hydrogel formulations (**r1**–**r4**), dynamic oscillatory amplitude sweeps were performed using an 8 mm parallel plate stainless steel geometry with a constant axial load of 100 mN, at a frequency of 1 Hz, and the strain was swept from 0.1 to 100%. Hydrogel discs were demolded after curing overnight at room temperature in a humid atmosphere and then incubated in PBS for 3 days to reach their equilibrium swelling. Storage moduli (*G*′), loss moduli (*G*″), and loss tangents (tan δ) were calculated. All measurements were performed at 25 °C.

### 2.9 Compressive Properties of Different Hydrogel Formulations

Compressive properties of hydrogel formulations (**r1**–**r4**) were assessed by using a mechanical tester (Shimadzu AGS-X universal, electromechanical) with a 10 N load cell. Samples were prepared in cylindrical molds (7 mm in diameter and 5 mm in height) and cured overnight in a humid atmosphere. Hydrogels were then demolded and incubated in PBS for 24 h before testing. A digital caliper was used to measure the dimensions of each hydrogel. Hydrogels were then compressed at a head speed of 1 mm/min until sample failure. The compressive modulus was determined by obtaining the slope of the linear region (0.01–0.1 mm/mm strain) of the stress (kPa) versus strain (mm/mm) curve.

### 2.10 Assessment of Gelation

Dynamic oscillatory time sweep was conducted at a frequency of 1 Hz and oscillation strain of 1% with a constant axial load of 100 mN using a 20 mm stainless steel parallel plate geometry at 25 and 37 °C. Approximately 500 μL of **r2** hydrogel was extruded onto the rheometer plate immediately after mixing of the components. Drying of the hydrogel samples was minimized using a rheometer solvent trap. Since the storage modulus could not reach a plateau until 44 h, a longer experiment was needed. However, sample drying turned out to be an issue even when the rheometer solvent trap was used for experiments longer than 44 h. Hence, **r2** hydrogel samples were cast and cured in the mold under a humid atmosphere for 1, 3, and 7 days at room temperature and measured at each time point by a rheological amplitude sweep at 1 Hz oscillation frequency while varying the strain from 0.1 to 100% at room temperature. The storage moduli at 1% strain, which is within the linear region for all samples, were compared to find out the time required for complete gelation.

### 2.11 Shear-Thinning

To evaluate the shear-thinning properties of **r2** hydrogels, viscosity was measured with an increasing shear rate from 0.01 to 100 s^–1^ in a continuous flow measurement under a constant axial load of 100 mN using a 20 mm stainless steel parallel plate geometry. Approximately 500 μL of **r2** hydrogels was extruded onto the rheometer plate immediately, 4 h, and 48 h after mixing the components. Measurements were performed at 25 °C.

### 2.12 Enzymatic Degradation

To evaluate enzymatic degradation *in vitro*, **r2** hydrogel was incubated for 1 h in Tris-HCl buffer (pH 7.4) at 37 °C. They were then placed in a vial containing preheated 5 U/mL collagenase solution in 0.1 M Tris-HCL (pH 7.4) and 5 mM CaCl_2_ and incubated at 37 °C. At indicated time points, hydrogels were weighed after blotting excess liquid using a filter paper, and the percentage of hydrogel mass remaining (*M*_*t*_) relative to the original swollen mass *M*_0_ was calculated  Fresh preheated collagenase solution was replaced for each sample at each time point.

### 2.13 Swelling of Hydrogel

To characterize the swelling, **r2** hydrogels were cut into 8 mm diameter discs, blotted with filter paper and their initial weights were recorded (*M*_0_). The samples were then incubated in 1 mL of PBS buffer at 37 °C for the indicated duration. At selected time intervals, the excess liquid was removed from the swollen gels, and the samples were reweighed (*M*_*t*_). Hydrogels were then replenished with a fresh buffer. From these data, swelling ratios  were calculated, and the equilibrium swelling ratio was recorded when the mass of the hydrogels no longer increased. The same experiment was also conducted at 25 °C for the indicated duration to assess the long-term storage stability of these hydrogels.

### 2.14 Self-Healing Properties of Hydrogel

To evaluate the self-healing properties of **r2** hydrogels after extrusion, hydrogel precursor components were first mixed in a syringe mixing system and kept for curing in the syringe for 48 h at room temperature. Afterward, the cross-linked hydrogel in the syringe was extruded and cast between two glass slides that were separated by a 1 mm spacer and cured overnight in a humid atmosphere and then stored in PBS for 3 days at room temperature. To study the viscoelastic properties of re-formed hydrogels, a dynamic oscillatory amplitude sweep was performed using an 8 mm parallel plate stainless steel geometry under a constant axial load of 100 mN at 1 Hz and compared with **r2** hydrogels that were extruded immediately from the syringe mixing system after mixing of the components, kept in the mold for 3 days, and then incubated in PBS for 3 days before being subjected to rheological amplitude sweep.

The gelation of **r2** hydrogels after extrusion was compared with the nonextruded sample. For this purpose, hydrogel precursor components were mixed in a syringe mixing system, kept for curing in the syringe for 48 h at room temperature, and then extruded directly onto the rheometer plate. A dynamic oscillatory time sweep was conducted at 1 Hz oscillation frequency, 1% oscillation strain, and 25 °C with a constant axial load of 100 mN using a 20 mm stainless steel parallel plate geometry and compared with a hydrogel that was extruded onto the rheometer plate directly after mixing of the components.

For the cysteine self-healing experiment, the **r2** hydrogel precursor components were first thoroughly mixed using a syringe mixing system and allowed to cure within the syringe for 48 h at room temperature. Following the curing process, the cross-linked hydrogel was extruded from the syringe. The self-healed sample was prepared by casting the extruded gel between two glass slides, separated by a 1 mm spacer, and allowing it to cure overnight in a humid environment. A second sample was treated with two equivalent amounts of cysteine relative to the furan groups. For this purpose, 50 μL of a 0.1 M solution of cysteine in PBS (pH 5) was added to 200 μL of extruded hydrogel before casting between two glass slides, also separated by a 1 mm spacer, and cured overnight in a humid atmosphere. Samples where cysteine was added did not form a gel whereas the no-cysteine samples formed hydrogel as usual.

^1^H NMR spectra were recorded for the original and extruded hydrogels. For this purpose, the hydrogels were degraded by collagenase in D_2_O. **r2** hydrogel precursor components were first mixed in a syringe mixing system and kept for curing in the syringe for 48 h at room temperature. Afterward, the cross-linked hydrogel in the syringe was injected using a 25 G needle. The injected sample was subsequently placed in a vial with a preheated collagenase solution (5 U/mL in D_2_O) and incubated at 37 °C. ^1^H NMR spectra of the digested solutions were recorded using a JEOL JNM-ECP series FT NMR spectrometer at a magnetic field strength of 9.4 T, operating at 400 MHz for ^1^H NMR. The experimental conditions were as follows: 400 MHz, 8000 scans, recorded at 25 °C and compared with **r2** hydrogels that were extruded immediately from the syringe mixing system after mixing the components, kept in the mold for 2 days, followed by incubation in preheated collagenase solution (5 U/mL in D_2_O) and incubated at 37 °C.

### 2.15 *In Vitro* Biocompatibility Studies

#### 2.15.1 Cell Culture

Human fetal cardiac mesenchymal stromal cells (hfcMSCs) and human umbilical vein endothelial cells (HUVECs; ATCC) were used for *in vitro* studies. hfcMSCs were cultured using MSC growth medium (MSC-GM, Lonza), supplemented with fetal bovine serum (10%) and penicillin–streptomycin (1%). HUVECs were maintained in endothelial basal medium (EBM-2, Lonza) enriched with endothelial growth factors (BulletKit, Lonza) and 100 units/mL penicillin–streptomycin (Gibco, USA). All cell cultures were maintained in a cell culture incubator (37 °C, 5% CO_2_). Cells were passaged approximately 2 times per week, and the medium was changed every second day.

#### 2.15.2 Cell Encapsulation

Collagen furan hydrogels (**r2**) were prepared according to the same protocol mentioned above except for using cell media instead of PBS. Cells were trypsinized and resuspended in a minimal amount of cell growth medium. For encapsulation experiments, cells (either hfcMSC or HUVEC) were mixed into the hydrogel by gentle pipetting to yield the final cell concentration in the hydrogel of 200 000 cells/200 μL and 300 000 cells/200 μL, respectively, and transferred into a syringe. Cell-laden hydrogels were cast into 48 well plates and cured for 1 h at 37 °C in a cell culture incubator, followed by adding the appropriate cell growth medium. The medium was changed every 2 days.

#### 2.15.3 Cell Viability

A commercial LIVE/DEAD viability/cytotoxicity kit (Invitrogen, Thermo Fisher, Sweden) was used to evaluate cell viability according to the instructions from the manufacturer. Cell viability was evaluated after 1 and 7 days of culture. Briefly, the culture medium was aspirated, and the hydrogels were washed with 1× sterile PBS and then incubated with 0.5 μL/mL of calcein AM and 2 μL/mL of ethidium homodimer in PBS for 45 min in the dark at 37 °C. Live cells were stained green, whereas dead cells were stained red. The cell-carrying hydrogels were imaged with a Zeiss LSM700 confocal microscope (Germany), and 3-D stacking and Z-sectioning was performed using LSM700 software. For each sample, at least five independent z-stacks at 10× magnification were analyzed. Cell viability was calculated as the ratio of live cells to the total number of cells using ImageJ software (*n* = 3).

#### 2.15.4 Cell Adhesion, Morphology, and Spreading

Fluorescent staining of F-actin filaments with Alexa Fluor 594 phalloidin (Invitrogen) and cell nuclei with 4,6-diamidino-2-phenylindole (DAPI; Sigma) was used to investigate attachment and spreading of encapsulated cells inside hydrogels. To stain the F-actin filaments of the cell, cell-laden hydrogels were fixed in 4% (v/v) paraformaldehyde (Sigma) for 30 min at room temperature and washed with PBS. The cells were then permeabilized in a 0.1% (w/v) Triton X-100 solution in PBS for 20 min and blocked in 1% (w/v) bovine serum albumin (BSA) for 1 h. The samples were then incubated in a solution of a 1:40 ratio of Alexa Fluor 594 phalloidin in 0.1% BSA for 45 min at room temperature to stain the actin cytoskeleton. For DAPI staining, the cell-laden hydrogels were incubated in a 0.1% (v/v) DAPI solution in PBS for 10 min at 37 °C to stain the nuclei. The stained samples were then washed three times with PBS before they were visualized with a Zeiss LSM700 confocal microscope (Germany).

### 2.16 *In Vivo* Biodegradation and Biocompatibility

#### 2.16.1 Dorsal Subcutaneous Implantation of Hydrogels

All animal protocols were approved by the Institutional Animal Care and Use Committee (IR.MUI.REC.1402.022) at Isfahan University. Male Wistar rats (200–250 g) were purchased from the Dental Research Center (Prof. Torabi Nejad Dentistry Research Center, Isfahan School of Dentistry, Isfahan, Iran) and kept in the animal core facility at Isfahan University (Water and Electrolyte Research Center, Isfahan University of Medical Sciences, Isfahan, Iran). Hydrogels were prepared under sterile conditions in cylindrical molds as described for compression testing, and their initial dry weights (day 0) were recorded after air drying. General anesthesia and analgesia were induced by intraperitoneal injection of 50 mg/kg of ketamine and 5 mg/kg of xylazine. Two 1 cm incisions were created on the posterior dorsomedial skin of the animals, and lateral subcutaneous pockets were prepared by blunt dissection. Dry hydrogels were then implanted into the subcutaneous pockets, followed by suturing and recovery from anesthesia.

#### 2.16.2 *In Vivo* Degradation and Histopathological Analysis

On days 5, 28, and 56 after implantations, the animals were euthanized by CO_2_ inhalation, and the samples were explanted for biodegradation and histopathological analysis. For biodegradation studies, the samples were carefully cleaned to remove the adjacent tissue and then washed three times with PBS and deionized water. Samples were then lyophilized to measure the weight loss over time.

For histological analysis, the surrounding tissues were fixed in 10% (v/v) formalin overnight. After fixation, the tissues were washed in running tap water for 24 h. Afterward, it was dehydrated in graded alcohols, cleared in xylene, and embedded in paraffin. 4 μm sections were taken from paraffin blocks with the help of a Leica RM2145 microtome. The slides were then subjected to hematoxylin and eosin (H&E) staining (Sigma) according to the manufacturer′s instructions. The examinations were carried out under the Olympus U-TV0.63XC microscope (Japan).

### 2.17 Statistical Analyses

All experiments were performed at least in triplicate, and data are presented as the mean ± standard deviation (SD). Statistical analysis was performed with GraphPad Prism software. One-way ANOVA one-way tests were performed to determine statistical significance, and *p* values less than 0.05 were considered as significant.

## 3 Results and Discussion

### 3.1 Synthesis and Characterization of Collagen Furan

**Figure 1:**
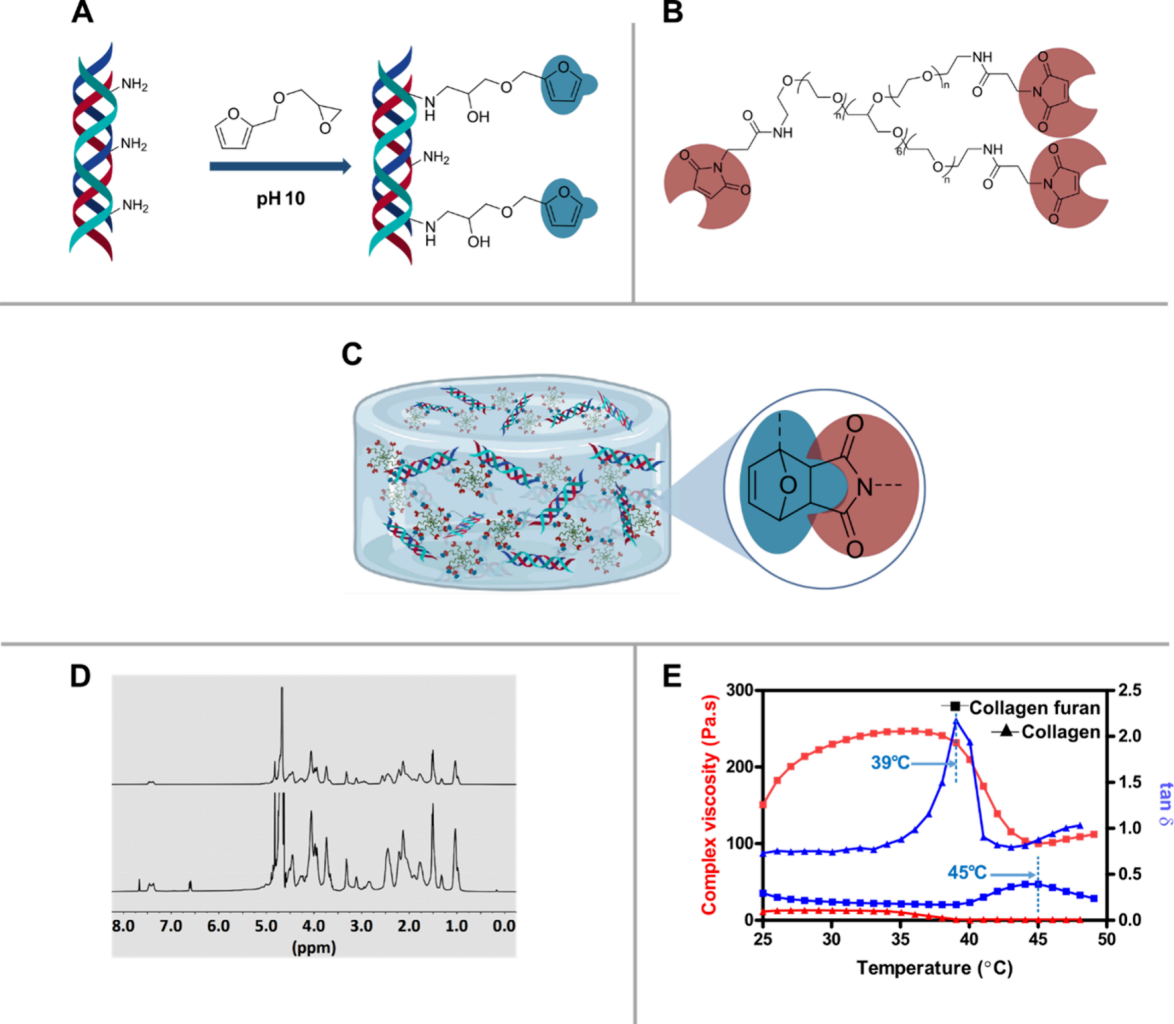
Overview of the collagen furan hydrogel. (A) Reaction scheme for the synthesis of collagen furan. (B) Chemical structure of the 8-arm polyethylene glycol maleimide (PEG-maleimide). (C) Schematic of collagen furan-PEG maleimide hydrogel with chemical structure of the cycloaddition product. (D) ^1^H NMR spectra in D_2_O of pristine collagen (top) and collagen furan (bottom). (E) Effect of temperature on the viscoelastic behaviors of 2% (w/v) collagen and collagen furan aqueous solutions.

The furan group was installed onto collagen through functionalization of the pendant amines from lysine and arginine side chains by reaction with furfuryl glycidyl ether (Figure 1A). The p*K*_a_ of these amines is 10.5, and therefore, the reaction was performed at pH 10 to ensure the deprotonated state of the amine for a nucleophilic ring opening reaction. Protein modification through pendant amines by reaction with glycidyl moieties is well-established. The resulting collagen furan (CF) was analyzed by ^1^H NMR spectroscopy, and the appearance of peaks at 6.38, 6.41, and 7.46 δ ppm was attributed to the aromatic protons of the furan group (Figure 1D). Additionally, collagen and collagen furan were further characterized by using FT-IR spectra (Figure S1). The degree of modification was determined by TNBS colorimetric assay and was found to be 61.4% ± 1.8% with respect to the amines in collagen. Moreover, circular dichroism spectroscopy was employed to ensure the structural integrity of the polyproline II triple helix in collagen furan. Both pristine collagen and collagen furan showed a negative ellipticity peak at 190 nm and a positive ellipticity peak at 220 nm which are characteristic of polyproline II triple helix (Figure S2).^52^ These data confirm the preservation of collagen triple helix in collagen furan.

Collagen is well-known for a lower critical solution temperature (LCST)-like transition and gel formation where the viscosity of collagen solution increases if the temperature is raised from 25 to 37 °C. This LCST-like transition is also responsible for collagen fibrillation at physiological pH and temperature. Temperatures higher than normal physiological temperature, however, cause melting of the collagen fibrillar assembly. Hence, a 2 wt % collagen furan solution has also been assessed for its capability of similar gel formation by oscillatory rheology where the complex viscosity (η*) was measured over a temperature range from 25 to 50 °C and compared with 2 wt % pristine collagen solution. Both pristine collagen and collagen furan showed a slight increase in η* by increasing the temperature from 25 °C and attained the highest value at 28 °C for pristine collagen and 36 °C for collagen furan, indicating fibrillation (Figure 1E). Further increase in temperature resulted in a decrease of complex viscosity, which reached a stable value at 45 °C for both pristine collagen and collagen furan. Moreover, the loss tangent (tan δ) increased with increasing temperature, indicating more damping and energy dissipation in the gel as can be expected due to the increased mobility of the individual collagen molecules within the intermolecular assembly at higher temperatures. The loss tangent reached its maxima at 39 and 45 °C for pristine collagen and collagen furan, respectively, indicative of the melting of the collagen triple helix at these temperatures.^53,54^ A higher triple helix melting temperature could be attributed to the stabilization of the triple helix rendered by the hydrophobic furan modifications in collagen furan. It was demonstrated earlier for collagen mimetic peptides that hydrophobic modifications bestow hyperstability in polyproline II triple helix.^55^ Moreover, above the melting temperature of the polyproline II triple helix, pristine collagen solution behaved as a viscous liquid with a tan δ higher than unity, whereas collagen furan still demonstrated a gel-like behavior with tan δ value residing below unity (Figure 1E). To our understanding, this is due to the hydrophobic associations between the furan groups on the random coil of collagen polypeptide after melting of the polyproline II triple helix.

### 3.2 Preparation of Hydrogel

To demonstrate the formation of chemically cross-linked hydrogel via cycloaddition between furan and maleimide groups, solutions of collagen furan and 8-arm PEG-maleimide in PBS were mixed at pH 5–6. Cross-linking was qualitatively confirmed by lifting the hydrogel from the mold with a tweezer or spatula.

**Figure 2:**
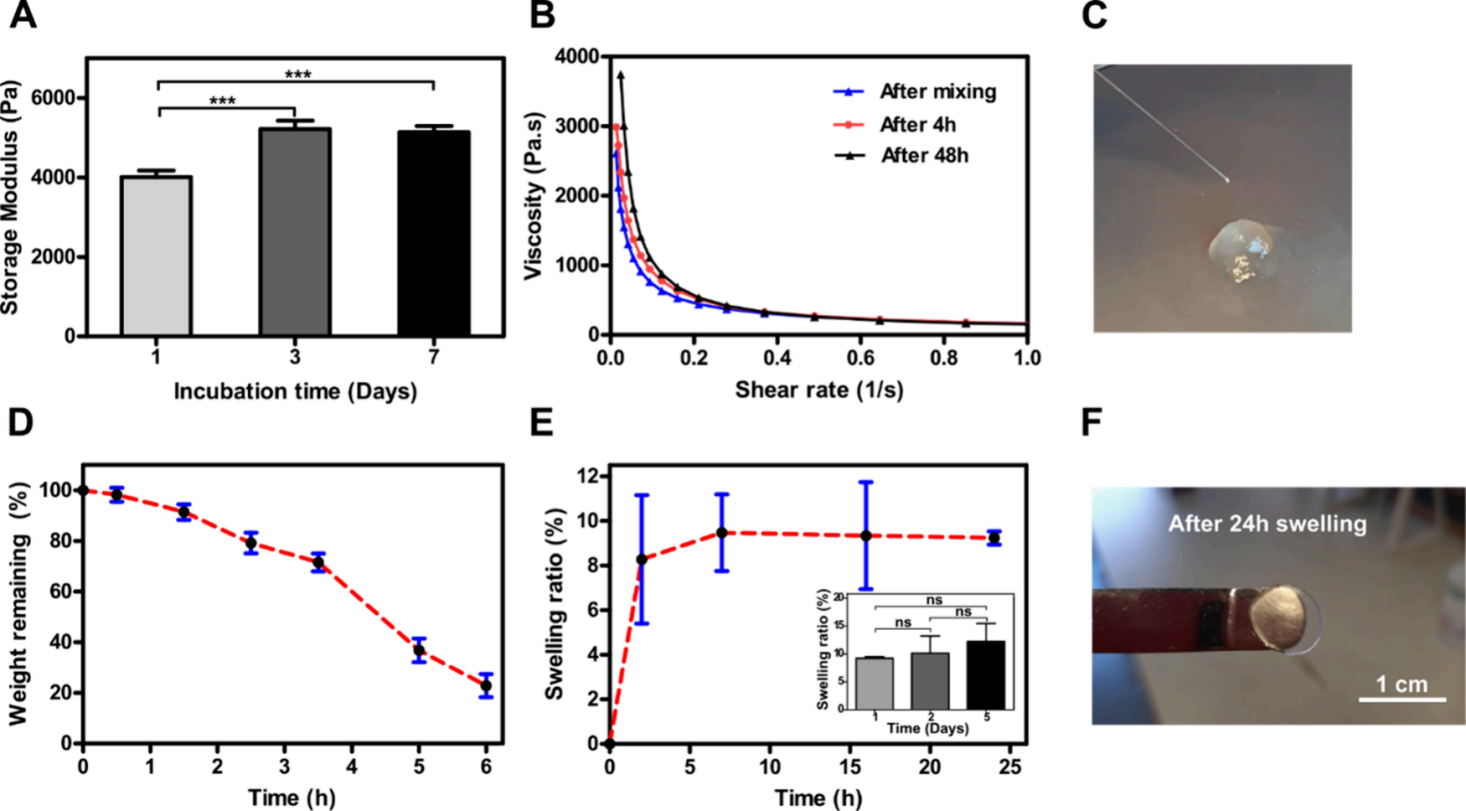
Assessment of gelation, shear-thinning, enzymatic degradation, and swelling properties of collagen furan hydrogels. (A) The storage moduli at 1% strain and 1 Hz oscillation frequency of **r2** hydrogel samples were compared at various time points to find out the time required for complete gelling (F_42.25_, df_6_). (B) Injectability and shear-thinning properties of collagen furan hydrogels; viscosity at increasing shear rate measured immediately, at 4 h, and at 48 h after hydrogel preparation. (C) Photograph of the injection experiment 48 h after hydrogel preparation from a 27 G needle. (D) Enzymatic degradation of collagen furan hydrogels in collagenase solution (5 U/mL), expressed as a percentage of initial weight remaining. (E) Swelling of collagen furan hydrogels stored in PBS at 37 °C for 24 h; inset, swelling over 5 days. (F) Photograph of collagen furan hydrogel after 24 h swelling (with the original shape of 8 mm diameter disc), scale bar = 1 cm. All experiments were performed with **r2** hydrogels (Table S1). Error bars represent standard deviation; “***” represents a *p*-value of ≤0.001; df represents degrees of freedom; one-way ANOVA, followed by Tukey’s test, *n* = 3 independent samples.

Furthermore, the time required for complete gelation was examined at 25 and 37 °C. For this purpose, the precursors of **r2** hydrogel (Supporting Information Table S1) were mixed and cast onto the rheometer plate, and the storage and loss moduli were monitored over 44 h (Figure S3). As non-cross-linked collagen solution is known to behave as a gel (*G*′ > *G*″) above 0.5% concentration at 1 Hz,^54^ a typical sol–gel transition was not possible to observe for collagen furan hydrogel formation. Rather the gradual increase of storage modulus over time was considered as gelling due to covalent cross-linking, and the time required to obtain a plateau in storage modulus was considered as the time needed for complete gelation. Nonetheless, the storage modulus of our developed hydrogel did not reach a plateau over 44 h (Figure S3). The rate of increase of storage modulus was slightly higher at 37 °C compared to 25 °C (Figure S3) as expected since the reaction can proceed faster at a higher temperature and collagen undergoes fibrillation, leading to gel formation at 37 °C. Hence, **r2** hydrogels were prepared separately and kept in the mold for different durations (1, 3, and 7 days) before subjecting them to rheological amplitude sweep for determination of storage and loss moduli. As shown in Figure 2A, storage modulus increased significantly with changing the curing time from 1 day to 3 days. In contrast, from day 3 to day 7, there was not a significant increase in the storage modulus.

### 3.3 Viscoelastic Properties of Collagen Furan Hydrogel

In an earlier study with chemically cross-linked injectable collagen hydrogel, we demonstrated that hydrogel moduli can be very easily fine-tuned by controlling the number of covalent cross-links formed by varying the stoichiometry between reaction partners.^29^ Although this is trivial for hydrogels prepared using synthetic polymer, such as polyethylene glycol, with low molecular weights where covalent cross-links are the sole determinants of elasticity,^56^ this is not the case for ECM-polymer based hydrogels where elasticity also originates from the self-assembly of polymer chains as well as chain entanglements. Earlier studies involving native telopeptide-rich collagen and matured telopeptide-poor collagen revealed more chain entanglements in the case of telopeptide-rich collagen.^57^ Since the collagen used in our studies is telopeptide-poor, it is rational to assume that there will not be many chain entanglements in the cross-linked hydrogels from collagen or from the PEG since the molecular weight of the PEG is low.^58^ However, the collagen will tend to assemble leading to elastic contribution and it could be impossible to fine-tune the collagen hydrogel moduli by only varying the covalent cross-links. This was found to be true in one of our earlier studies.^27^ However, in an attempt to fine-tune the hydrogel moduli, different stoichiometric ratios of furan to maleimide were investigated using oscillatory rheology (Figure S4). With increasing the furan to maleimide ratio from 1 to 2, the *G*′ (3245 ± 218 Pa vs 4010 ± 165 Pa) and *G*″ (380 ± 23 Pa vs 463 ± 37 Pa) increased further (*p* < 0.05); however, further increasing of maleimide to furan ratio has no significant effect on *G*′ and *G*″ values, (Figure S4B,C). The loss tangents were found to have no statistically significant differences among various formulations (Figure S4D).

### 3.4 Compression Properties of Collagen Furan Hydrogels

To test the mechanical properties of the collagen-furan hydrogels, unconfined compression was performed on different hydrogel formulations with varying furan:maleimide ratios (**r1**–**r4**) (Figure S5). The **r2** hydrogel was demonstrated to have a compressive modulus of 7.42 ± 0.94 kPa which was found to be significantly higher than the **r1** hydrogel (Figure S5A). However, no statistically significant differences could be observed for compressive strength and strain among all formulations (Figure S5B,C). All following experiments were performed using the **r2** hydrogel.

### 3.5 Injectability and Shear-Thinning Properties of Collagen Furan Hydrogel

To evaluate the injectability and shear-thinning properties of hydrogels, all components of **r2** hydrogel were mixed in the syringe mixing system, and the cross-linking reaction was allowed to proceed in the syringe for different periods (0, 4, and 48 h), followed by extrusion through a 27 G needle from the syringe onto the rheometer plate. Afterward, viscosity under continuous shear was measured by increasing the shear rate from 0.01 to 100 s^–1^ as shown in Figure 2B.

In an earlier study involving a synthetic polymer network for the preparation of elastomers and thermosets, the furan-maleimide cycloadduct was reported to undergo cycloreversion at high temperatures.^59^ To our understanding, the shear force generated during injection at 25 °C acts as a force-actuator mimicking elevated temperature resulting in mechanochemical decoupling of the cycloadduct.^60−63^ Furthermore, these hydrogels were injectable at least up to 48 h after mixing the precursors when stored at room temperature (Figure 2B,C).

### 3.6 Enzymatic Degradation and Swelling of Hydrogel

To evaluate enzymatic degradation, hydrogels were incubated in collagenase solution *in vitro*; as a result, the hydrogels were degraded to a degree that made it impossible to weigh them after 6 h and completely dissociated soon after (Figure 2D).

For clinical applications, where the gel is injected into the tissue defect to fill the cavity, it is important that the injected hydrogel does not swell too much, as prolonged swelling will cause enhanced pressure on the tissue around the injection site and risk the hydrogel popping out of the defect. On the other hand, poor mechanical strength after swelling has always been a major disadvantage of injectable hydrogels. In this study, the swelling ratio (wet/wet swelling ratio) was measured by evaluating the equilibrium hydrogel weight after 24 h of swelling in PBS at 37 °C to as prepared weight determined immediately after cross-linking to understand how much collagen furan hydrogel might swell after injection and cross-linking. As shown in Figure 2E, the collagen furan hydrogel showed a minor swelling degree of 10% after 24 h from the as-prepared state. This small extent of swelling was not visible in observations by the naked eye (Figure 2F). As minor swelling was observed, a swelling experiment over 5 days was performed in a similar manner which revealed no significant swelling when compared to 24 h of swelling (Figure 2E inset). Moreover, to determine the long-term storage stability of these hydrogels, a swelling experiment was performed in PBS at 25 °C for 28 days (Figure S6), and no significant swelling was observed. By introducing hydrophobic furan to the collagen polymer chain and further cross-linking with the maleimide group, the overall hydrophilicity of the polymer network decreases with a concomitant increase in the elasticity of the network, leading to lower swelling.

### 3.7 Autonomous Self-Healing after Extrusion

**Figure 3:**
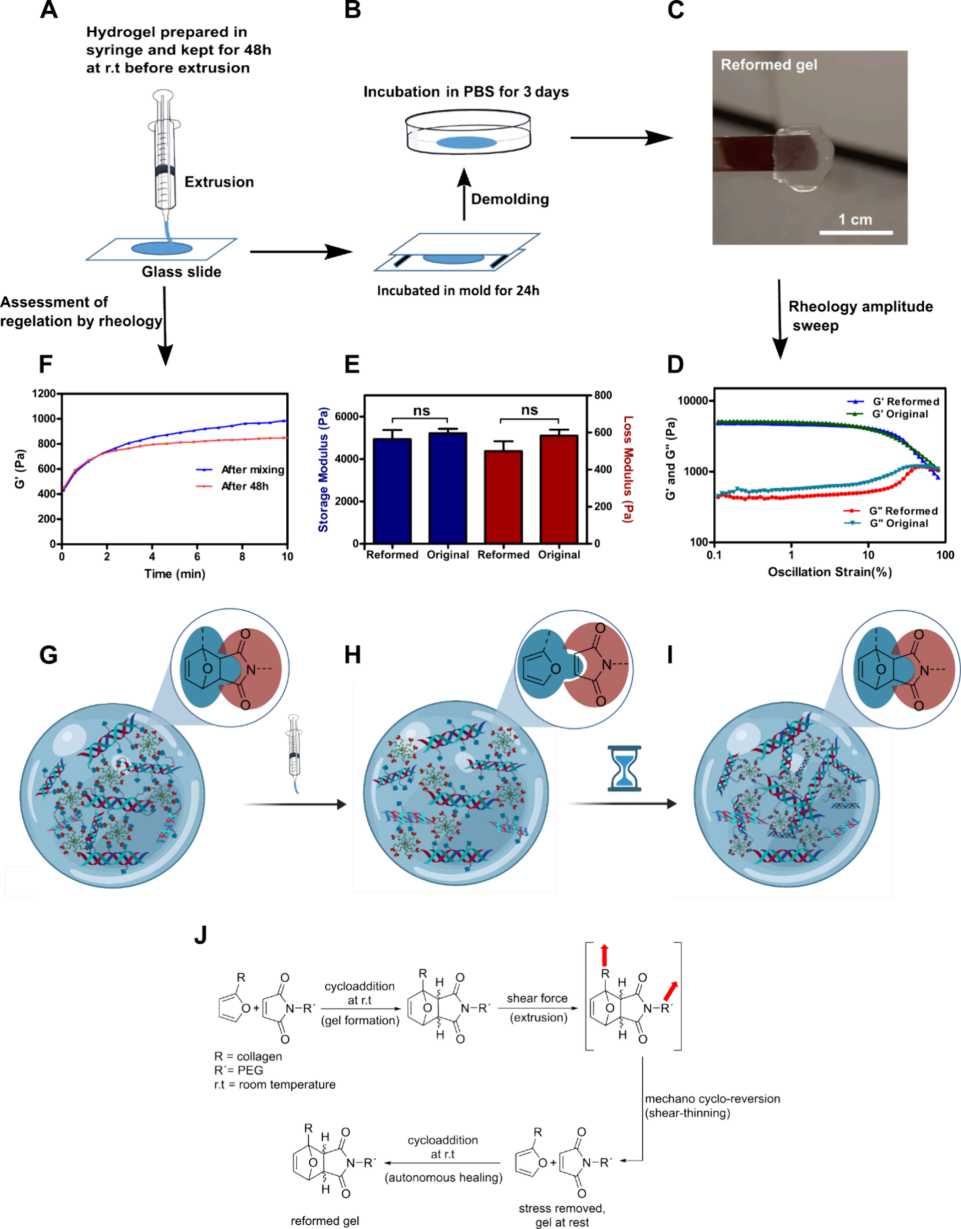
Autonomous self-healing properties of collagen furan hydrogels. (A, B) Schematic of the self-healing experiment. (C) Photograph of the re-formed hydrogel. (D) Representative dynamic oscillatory amplitude sweep of re-formed hydrogels compared with original hydrogels that were kept in the mold for 3 days, followed by incubation in PBS for 3 days. (E) The storage and loss moduli at 1% strain of re-formed hydrogels compared to original samples (F_389.3_, df_15_). (F) Assessment of gelation by rheology time sweep immediately after mixing of precursors and at extrusion after 48 h incubation in the syringe as depicted in (A). (G–I) Schematic representation of shear-stress-induced cycloreversion during injection followed by cycloaddition after the stress is removed, leading to shear-thinning and autonomous self-healing, respectively. (J) Chemical structures of plausible intermediates formed during shear-thinning and autonomous self-healing (red arrows indicate stress). All experiments were performed with **r2** hydrogels. Error bars represent standard deviation; “ns” represents nonsignificant; df represents degrees of freedom; one-way ANOVA, followed by Tukey’s test, *n* = 3 independent samples.

To evaluate the self-healing of the developed hydrogels after extrusion, all components were mixed and kept in the syringe mixing system for 48 h at room temperature (rt) for the cross-linking reaction to proceed. Afterward, the formed hydrogel was extruded (Figure 3A) onto a glass slide and incubated in the mold for 24 h at rt (Figure 3B). Then, the hydrogel was demolded and incubated in PBS for 3 days at rt (Figure 3B) followed by rheology amplitude-sweep to determine the modulus and linear viscoelastic region of the re-formed hydrogel (Figure 3C–E). In a parallel experiment, the extruded hydrogel as described in Figure 3A was subjected to rheology time-sweep for the assessment of the rate of regelation (Figure 3F). The extruded hydrogels were re-formed without needing any external trigger, resulting in a homogeneous hydrogel that could be picked up by a spatula (Figure 3C). To further quantitatively assess the self-healing of the extruded hydrogels, oscillatory amplitude sweeps were performed and compared side-by-side with the original hydrogels (Figure 3D,E). To our surprise, no statistically significant differences could be observed between original and re-formed samples in terms of storage and loss moduli (Figure 3E), and the graph of *G*′ vs oscillation strain was found to be nearly identical for both original and re-formed hydrogels (Figure 3D). This led us to conclude that the self-healing is quantitative in terms of stiffness, indicated by *G*′, and the linear viscoelastic region, indicated by the *G*′ versus oscillation strain graph. Moreover, time-sweep rheology revealed that the rate of regelation of the extruded hydrogels was nearly identical to that of the original hydrogel (Figure 3F). To our understanding, the shear force during extrusion acted as a trigger for the cycloreversion of the furan-maleimide cycloadduct and resulted in the shear-thinning behavior of this hydrogel (Figure 3G). However, after extrusion when the hydrogel was at rest and no stress was applied (Figure 3H), the furan and the maleimide functional groups could slowly react resulting in the observed self-healing (Figure 3I). Notably, the near-identical initial rate of gelation for the original and the extruded hydrogels further indicates force-induced cycloreversion of the furan-maleimide cycloadduct, as a similar rate of gelation could not have been otherwise explained by considering a reaction between unreacted furan and maleimide groups from the first-time reaction during hydrogel formation.

To support this hypothesis, cysteine was added to the extruded hydrogels and compared with the same, but where no cysteine was added. No hydrogels were formed for the cysteine samples, whereas gels could be formed with the no-cysteine samples. Additionally, original and extruded hydrogels were digested with collagenase, and ^1^H NMR spectra were recorded. Several peaks corroborating the presence of furan and maleimide functional groups could be observed in the extruded samples, whereas no such peaks were found in the original samples (Figure S7).

The cycloaddition reaction between furan and maleimide can yield a kinetically controlled *endo* and a thermodynamically controlled *exo* adduct. The cycloreversion at higher temperatures can also be explained considering the forward reaction (cycloaddition) being entropically unfavorable. In the case of hydrogels prepared using this reaction, high temperatures and relatively long incubation times were needed to obtain self-healing from a single incision.^42^ To our understanding, this is indicative of the thermodynamically controlled furan-maleimide cycloaddition resulting in the observed temperature-triggered self-healing behavior.

Cycloreversion of furan-maleimide cycloadduct under mechanical force has so far been only observed in the case of single polymer chains.^61−63^ The mechanical stretching was provided using ultrasound. Proximal cycloadducts, such as we have in the system, have been demonstrated to undergo cycloreversion under mechanical stretching.^61^ In a recent study, researchers have demonstrated that such cycloreversion under tension may follow a pericyclic or a nonpericyclic pathway depending on the amount of applied force.^60^ Nonetheless, such cycloreversion induced by shear stress has not been observed so far in ECM-polymer-derived hydrogel. Plausible chemical structures involved in this process are depicted in Figure 3J. Although it appears to be intuitive that having mechanophores within cross-links will result in a hydrogel being self-healing, this is a vast oversimplification. Several studies in this field debate and discuss the difficulties in activating a mechanophore in a cross-linked hydrogel system where the applied macroscopic stress was never distributed homogeneously over the complete cross-linked network and then funneled to the mechanophores.^64^ In fact, only a tiny fraction of the mechanophores is usually activated through applied macroscopic stress. To our surprise, the shear-thinning and autonomous self-healing behaviors of the hydrogels developed here indicate mechanophore activation by using macroscopic stress. To the best of our knowledge, this is the only example of a hydrogel of this kind. Although intriguing, it should be noted that rheology is a macroscopic evaluation technique, and apparent complete macroscopic autonomous healing in terms of rate of gelation, moduli, or linear viscoelastic limit does not necessarily imply complete healing at the microscale. More studies, especially those that can reveal microscale structural information under stress, could be great tools to explore this further, which would be instrumental in rationally designing such hydrogel systems. Nevertheless, ECM-derived injectable hydrogels with shear-thinning and autonomous self-healing, shape-fidelity, and nonswelling over the long term are a major leap forward in the field of injectable biomaterials.

### 3.8 *In Vitro* Biological Characterization of Hydrogels

**Figure 4:**
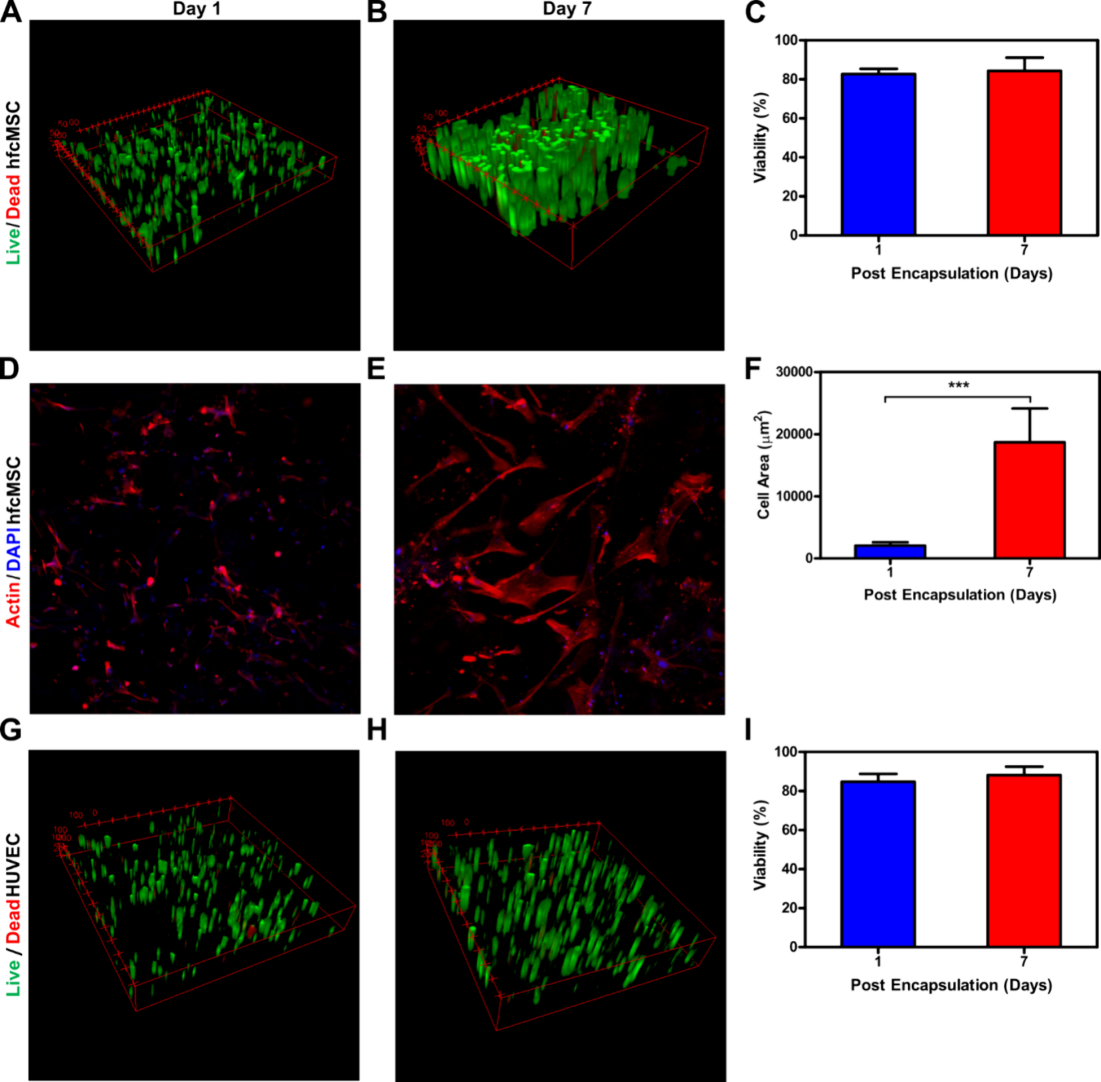
*In vitro* 3D cultures of hfcMSCs and HUVECs encapsulated in collagen furan hydrogels. (A) Representative live/dead 3D scans of hfcMSCs 1 day and (B) 7 days after encapsulation. (C) Percentage of alive hfcMSCs for up to 7 days in 3D culture with quantification of the viability images. (D) Representative phalloidin (red)/DAPI (blue) stained images of hfcMSCs 1 day and (E) 7 days after encapsulation. (F) Quantification of the hfcMSCs area encapsulated in hydrogels after 1 and 7 days (F_108.9_, df_26_). (G) Representative 3D scans of HUVECs, using live/dead assay 1 day and (H) 7 days after encapsulation. (I) Percentage of live HUVECs for up to 7 days in 3D culture quantification of viability images. All experiments were performed with **r2** hydrogels. Error bars represent standard deviation; “***” represents a *p*-value of ≤0.001; df represents degrees of freedom; one-way ANOVA, followed by Tukey’s test, *n* = 3 independent samples.

To investigate the impact of cross-linking chemistry on cell viability, we conducted an *in vitro* assessment of cytocompatibility for **r2** hydrogels. Human fetal cardiac mesenchymal stromal cells (hfcMSCs) and human umbilical vein endothelial cell lines (HUVECs) were employed as model cells encapsulated within these hydrogels. We evaluated cell viability at 1 day and extended it to 7 days postencapsulation using the calcein-AM/ethidium homodimer live/dead assay, examining the fate of both hfcMSCs (Figure 4A–C) and HUVECs (Figure 4G–I).

Our findings reveal that cell viability consistently exceeded 80% for the developed hydrogels even up to 7 days after cell encapsulation. This robust viability suggests that the engineered collagen furan hydrogels exhibited no cytotoxicity toward either hfcMSCs or HUVECs. These results align with prior studies demonstrating that the Diels–Alder reaction, even during cross-linking, occurs under mild conditions and does not exert any toxicity on cells. Combined with their shear-thinning and self-healing properties, these results demonstrated the capacity of developed collagen furan hydrogels to act as a cargo for cell delivery. Moreover, cell morphology (adhesion and spreading) was assessed by fixing the cell-laden hydrogels (hfcMSCs) and staining the actin cytoskeleton with fluorescent phalloidin and nuclei with DAPI. Cell spreading in collagen furan hydrogels was evaluated by measuring the cell area under a microscope. As shown in Figure 4D, hfcMSCs adhered effectively to the three-dimensional hydrogel structure even 1 day after encapsulation. After 7 days, we observed a noticeable increase in cell adhesion and localized remodeling of the hydrogel, as evidenced by the hfcMSCs significantly spreading into the surrounding material (Figure 4D,E). Consequently, the quantified data in Figure 4F substantiate that the cell area had significantly increased.

### 3.9 Biocompatibility and Biodegradation of the Hydrogels *in Vivo*

To assess the suitability of collagen-furan hydrogels for tissue engineering applications, *in vivo* biodegradation and biocompatibility were investigated by using a rat subcutaneous implantation model. Cylindrical hydrogel samples (7 mm in diameter and 2 mm in height) were prepared using formulation **r2**, lyophilized, and their weights were recorded. These dried samples were subsequently implanted subcutaneously into the dorsal dorsal of male Wistar rats. A total of six rats were involved in the study, with two rats per time point and two samples per rat. After 5, 28, and 56 days postimplantation, the rats were euthanized using CO_2_ inhalation, and the samples were extracted for analysis of biodegradation and histopathology.

**Figure 5:**
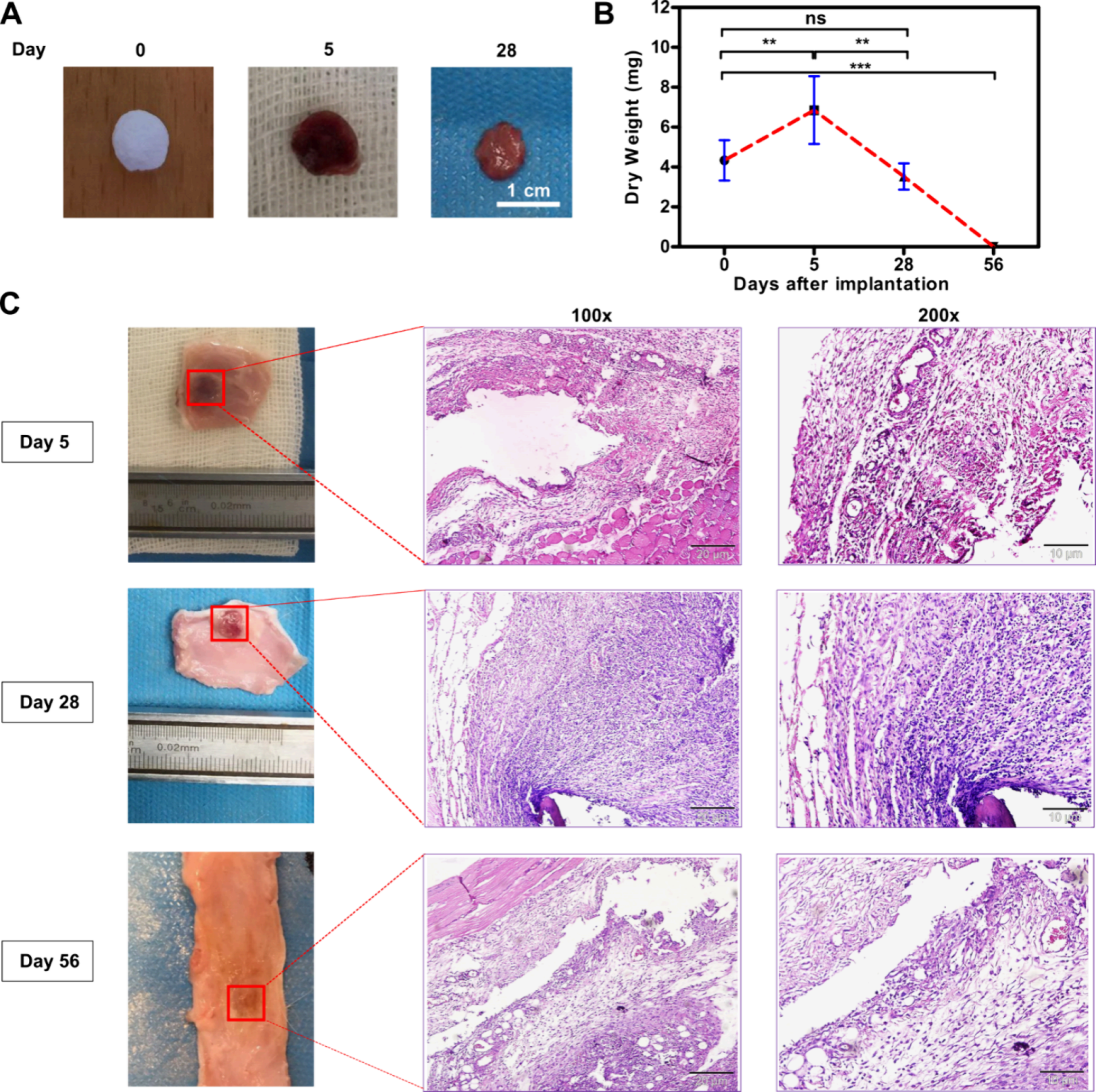
*In vivo* biocompatibility and biodegradation of collagen furan hydrogels using a rat subcutaneous implantation model. (A) Representative images of collagen furan scaffold before implantation (day 0) and on days 5 and 28 postimplantation. (B) *In vivo* biodegradation of collagen furan scaffolds on days 0, 5, 28, and 56 postimplantations (F_30.9_, df_23_). (C) Hydrogels with the surrounding tissue after 5, 28, and 56 days of implantation and hematoxylin and eosin (H&E) staining of tissue sections (100× and 200× with scale bars 20 and 10 μm). All experiments were performed with **r2** hydrogels. Error bars represent standard deviation; “**” represents a *p*-value of ≤0.01; “***” represents a *p*-value of ≤0.001; “ns” represents nonsignificant; df represents degrees of freedom; one-way ANOVA, followed by Tukey’s test, *n* = 3 independent samples.

The explants were cleansed with PBS and deionized water, lyophilized, weighed, and subjected to direct visual inspection. Histological staining was performed on the adjacent tissue. Visual examination of the explanted samples revealed evidence of blood penetration and clotting, as indicated by a change in the color to red. Additionally, the physical size of the samples decreased from day 5 to day 28, ultimately leading to complete resorption by day 56 (Figure 5A). As depicted in Figure 5B, the average dry weight of the explants showed a significant increase from 4.3 ± 1.0 mg to 6.85 ± 1.7 mg after 5 days of implantation, likely attributable to blood penetration and clotting. Subsequently, their weight decreased significantly to 3.5 ± 0.6 mg at day 28, indicative of *in vivo* biodegradation, ultimately resulting in complete resorption by day 56 postimplantation.

Furthermore, the biocompatibility of collagen-furan samples was assessed via a histological analysis of subcutaneously implanted samples (Figure 5C). Hematoxylin and eosin (H&E) staining revealed slight to mild accumulation of lymphocytes in the adjacent tissues of samples at day 5, moderate inflammation at day 28, and rare scattered lymphocytes at day 56 postimplantation. However, no other inflammatory factors, such as giant cells or macrophages, were observed at any time point. Staining also indicated that the mean rate of fibrosis was mild at day 5, with no apparent signs of fibrous tissue formation at day 28 and day 56. The mean rate of edema was mild on day 5 and completely disappeared on days 28 and 56. Notably, granulation tissue was absent at all time points, and congestion was only observed on day 5. Integration between the samples and their adjacent tissues was visually apparent in all rats at all time points and further confirmed by staining on day 5 (Figure S8).

Subcutaneous implantation is a widely used model for biocompatibility and degradation studies due to its accessibility, ease of monitoring, and relatively straightforward surgical procedure. It allows for the direct observation of material–tissue interactions, making it an excellent model for assessing the immune response, fibrosis, and general tissue compatibility. Additionally, subcutaneous tissue provides a controlled environment with fewer variables than more complex implantation sites, such as joints or vascular tissues, allowing for clearer insights into the material’s behavior.^65,66^ However, this simplicity can also be a limitation, as it may not fully replicate the mechanical stresses, blood flow, or cell types found in other anatomical locations.^67^ Additionally, this test may not accurately predict long-term biological responses and potential systemic effects due to its localized nature.^68^

*In vivo*, the complex environment, including mechanical stresses and dynamic interactions with the immune system, results in a gradual and progressive degradation over time.^69^ This is evident in our subcutaneous implantation study, where the material degraded over approximately 56 days. The presence of recruited cells, such as fibroblasts and macrophages, at the implant site, contributes to localized MMP production, further slowing the degradation process.^70^ In contrast, the 5 U/mL of collagenase was used in our study to accelerate the degradation process for faster analysis. *In vitro*, the material degraded within 6 h, much faster than *in vivo*. This discrepancy is due to the higher and more uniform collagenase activity *in vitro*, which lacks the regulatory elements, such as TIMPs and cellular interactions, present in a living system.

In summary, the results suggest that collagen furan hydrogels exhibit high *in vivo* biocompatibility, as made evident by the absence of sustained inflammatory responses in the host organism. These findings collectively support the potential use of collagen furan hydrogels in biomedical applications, owing to their excellent biocompatibility and biodegradation characteristics. However, given the limitations of subcutaneous implantation study as described above, testing these hydrogels in specific *in vivo* models tailored toward particular medical conditions is necessary to fully realize the potential of these hydrogels.

## 4 Conclusions

Herein we developed a collagen-based injectable hydrogel cross-linked using a bio-orthogonal cycloaddition reaction between furan and maleimide. The furan-collagen developed here has thermal gelling properties similar to those of pristine collagen but is more stable upon storage compared to previously developed thiol-collagens developed for similar purposes. The resulting cross-linked hydrogel is shear-thinning, does not swell or shrink, can retain its shape and size even after prolonged storage in buffer, can be degraded by enzymes *in vitro*, and is completely resorbable *in vivo*. The developed hydrogels can be injected in the gel state and retain their injectability and shear-thinning properties up to 48 h after mixing the hydrogel components. This is a significant leap forward in the field of *in vivo* injectable ECM-polymer-derived hydrogels since the hydrogel can be prepared in a sterile environment outside the operation theater and then brought to the surgical room at a relevant time point. Moreover, the injected hydrogel is capable of quantitatively autonomous self-healing after injection. The existing literature on self-healing hydrogels primarily focuses on the recovery of hydrogels in terms of moduli (stiffness) after a shear stress is applied, and very little endeavors are dedicated to assessing the recovery of other crucial viscoelastic parameters such as the linear viscoelastic limit, yield strength, stored and dissipated energy at varying strains, or kinetics of regelation. Moreover, in most reported cases, the shear stress from an injection is simulated by applying a single incision where the spread of the damaged zone in the hydrogel is quite limited. In contrast, a hydrogel experiences a significantly higher strain and shear rate over the whole sample during injection through a needle. The developed hydrogel can quantitatively recover, without requiring any external trigger, from such an injection shear stress not only in terms of moduli but also regarding the linear viscoelastic limit and, thereby, yield strength and stored and dissipated energy at varying strains. To our surprise, even the kinetics of regelation (cross-linking) turned out to be the same as that of gelation of virgin (noninjected) samples. The observed shear-thinning and autonomous self-healing behaviors were explained by the stress-induced cycloreversion of the cycloadduct cross-links followed by cycloaddition between the reacting partners to re-form the gel after injection. This is the first example of an injectable collagen hydrogel that can undergo a complete autonomous self-healing after injection in terms of several viscoelastic parameters. Due to the challenges involved in mechanophore activation within a cross-linked polymer network by applying macroscopic stress, supramolecular interactions or reversible cross-linking reactions are often thought to be better choices for designing self-healing hydrogels. However, such hydrogel systems suffer from the risk of swelling in the buffer or require external triggers to favor the reverse reaction during the self-healing process. Furthermore, *in vitro* and *in vivo* experiments with cells and animal models further demonstrate the usability of the developed collagen furan hydrogels in regenerative medicine. To our knowledge, this is the first example of an injectable, shape-holding collagen hydrogel that undergoes complete autonomous self-healing where cell encapsulation is possible.

The findings of this study present both significant insights and some limitations that warrant further investigation. One key observation is that the viscoelastic properties of the hydrogels could not be fine-tuned by altering the reactant stoichiometry. This, coupled with the fact that the hydrogels appeared opaque, suggests the formation of nonhomogeneous hydrogels. These factors limit the current application potential of the hydrogels. A potential solution to this problem is to reduce the degree of furan modifications or employ a more water-soluble furan derivative. Although the first approach may appear appealing at first glance, it may result in hydrogels that will swell due to the reduced number of cross-links formed. The second approach, however, would require the design and synthesis of a new small-molecule furan derivative that is more hydrophilic than commercially available furan derivatives, which are highly hydrophobic and could be used for collagen modification. We demonstrated earlier that even for ECM-polymer-based hydrogels if perfect network homogeneity is obtained, it is possible to fine-tune the viscoelastic properties by altering reacting group stoichiometry^29^ akin to synthetic polymer networks.^56^ Such control on viscoelastic properties would be crucial for broadening the applicability of hydrogel biomaterials in fields such as regenerative medicine and drug delivery.

Another important limitation is the uncertainty surrounding the self-healing properties of the hydrogels over time. In this study, self-healing was observed in samples extruded after 2 days of mixing the precursor components. However, it remains unknown whether this self-healing capability will persist beyond this time point or if complete healing, rather than partial healing, can be observed at later time points as in one of our earlier reports.^29^ Addressing this issue will be a key focus of future studies.

Additionally, the underlying mechanism by which macroscopic shear stress is funneled down to microscopic mechanophore cross-links remains unclear. Understanding this process is critical to improving the design and function of these hydrogels. To address this gap, we plan to conduct small-angle neutron scattering (SANS) and ultrasmall-angle neutron scattering (USANS) experiments on the hydrogels both at rest and under stress. These experiments will allow us to observe structural changes at the microscale under stress, which could provide critical insights into the relationship between the macroscopic mechanical properties and microscale architecture. Such knowledge will be instrumental in guiding the rational design of future hydrogel systems.

The potential application of such materials could be in tissue-bulking of the left-ventricular wall after myocardial infarction to prevent further dilation of the heart coupled with the sustained release of encapsulated drugs where shear-stress from heart pulsation would facilitate the release of the encapsulated drug but autonomous self-healing would recover the strength of the material, and hence, the mechanical reinforcement of the ventricular wall will not be compromised during the drug release. Notably, for future clinical trials, it is essential not only to acquire data in more specific *in vivo* models but also to address the challenges of scaling up during GMP production, aseptic handling, and the use of xenogeneic proteins.

## Supporting Information Available

FT-IR spectra in Figure S1, circular dichroism spectra in Figure S2, assessment of gelation in Figure S3, oscillatory amplitude sweep of all hydrogel formulations in Figure S4, unconfined compression of all hydrogel formulations in Figure S5, swelling in Figure S6, ^1^H NMR spectra in Figure S7, H&E staining of hydrogel explants in Figure S8, composition of all hydrogel formulations in Table S1 (PDF)
