# Supplementary material for: Shear-Induced Cycloreversion Leading to Shear-Thinning and Autonomous Self-Healing in an Injectable, Shape-Holding Collagen Hydrogel
Source: ACS Appl Mater Interfaces. 2024 Oct 8;16(41):55056–70. doi: 10.1021/acsami.4c08066 (PMC11492320; doi:10.1021/acsami.4c08066)
Supplement: Supplementary file 1 — am4c08066_si_001.pdf [file am4c08066_si_001.pdf]

Supporting Information for

Shear-Induced Cyclo-Reversion Leading to Shear-Thinning and Autonomous Self-Healing in an Injectable, Shape-Holding Collagen Hydrogel

Mahsa Jamadi Khiabani,[1] Sareh Soroushzadeh,[2] Ardeshir Talebi,[2] and Ayan Samanta*[, 1]

[1]Macromolecular Chemistry, Department of Chemistry – Ångström Laboratory, Uppsala University, Box 538, 751 21 Uppsala, Sweden

[2] Department of Pathology, School of Medicine, Isfahan University of Medical Sciences, Isfahan, Iran

*Corresponding author: ayan.samanta@kemi.uu.se

Page S1. This page

Page S2-S6. Figures S1-S8, Table S1

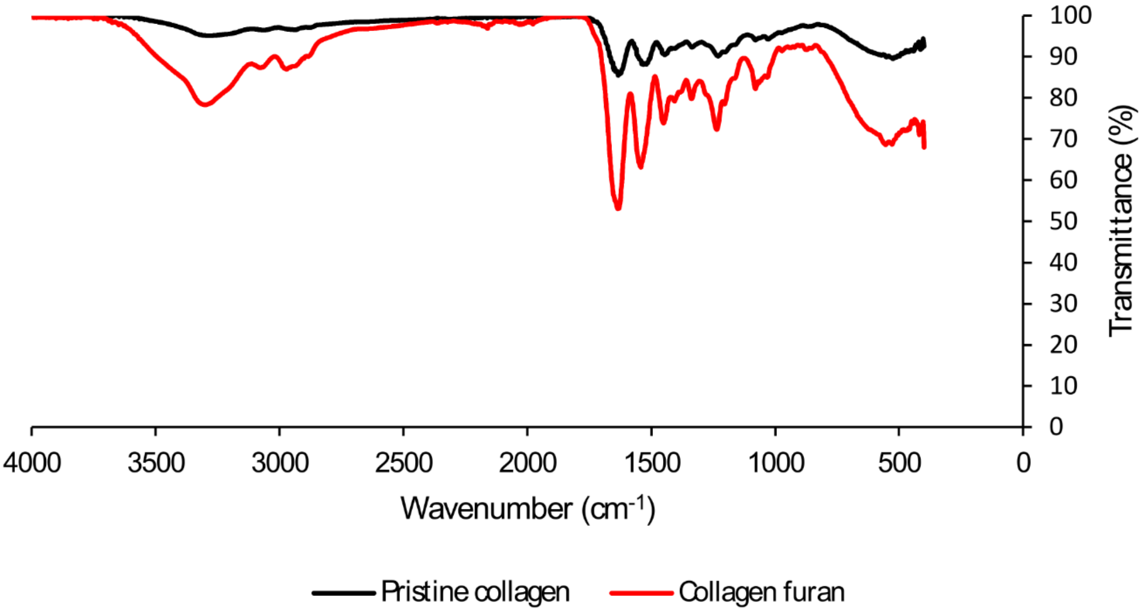

Figure S1. FT-IR spectra of pristine collagen and collagen furan.

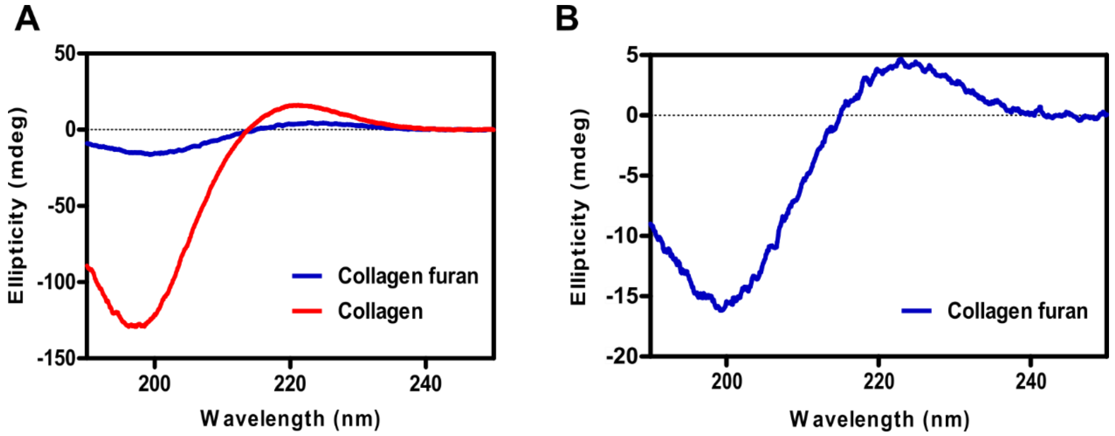

Figure S2. Circular dichroism spectra; **A:** collagen furan and pristine collagen, **B:** magnified view of collagen furan.

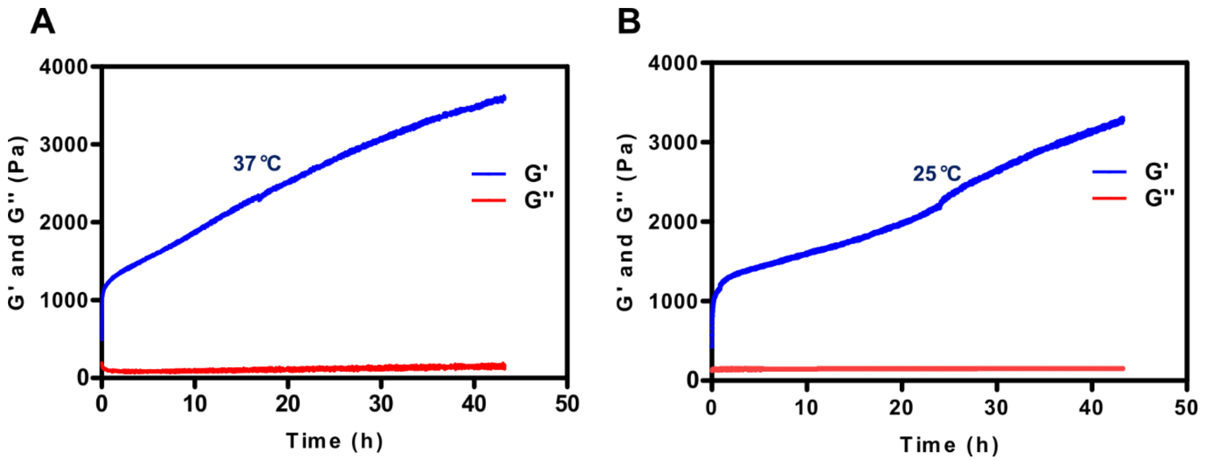

Figure S3. Dynamic oscillatory time sweep to monitor gelation; **A:** experiment conducted at 37 °C, **B:** experiment conducted at 25 °C. Formulation used **r2**.

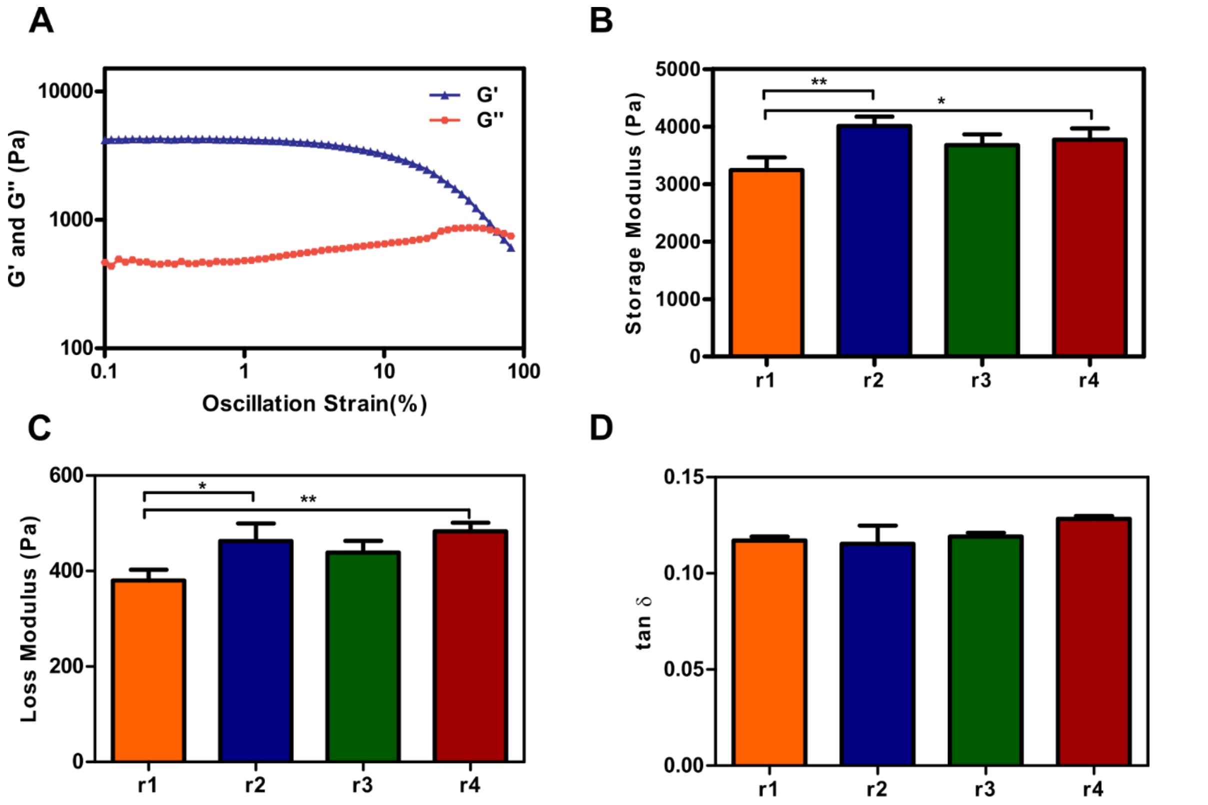

Figure S4. Characterization of the viscoelastic properties of collagen furan hydrogels (**r1** - **r4**); **A:** representative dynamic oscillatory amplitude sweep of **r2** hydrogel, **B:** storage moduli of all hydrogels (**r1** - **r4**) at 1 Hz oscillation frequency and 1% oscillation strain ($F_{8.279}$, df_{11}), **C:** loss moduli of all hydrogels (**r1** - **r4**) at 1 Hz oscillation frequency and 1% oscillation strain ($F_{8.518}$, df_{11}), **D:** loss tangent of all hydrogels (**r1** - **r4**) at 1 Hz oscillation frequency and 1% oscillation strain . All measurements were performed at 25 °C. Error bars represent standard deviation, "*" represents a p-value of ≤0.05, "**" represents a p-value of ≤0.01; df represents degrees of freedom; one-way ANOVA, followed by Tukey's test, n = 3 independent samples.

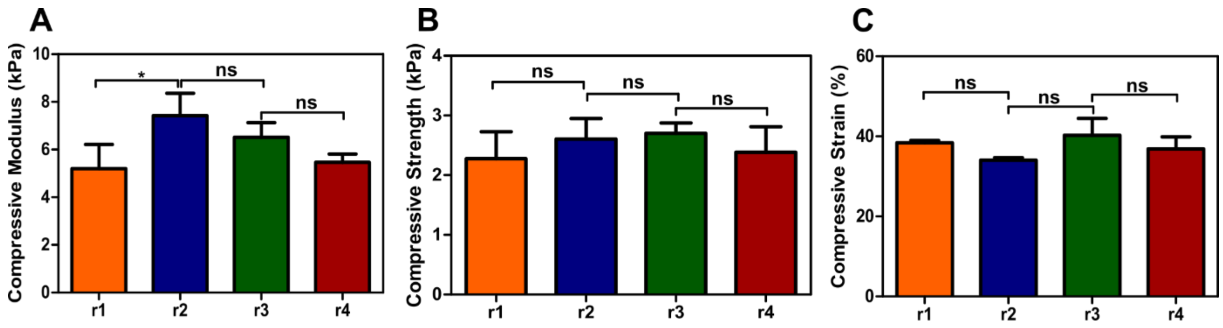

Figure S5. Unconfined compression of collagen furan hydrogels (**r1 - r4**); **A:** compressive moduli derived from the linear region (0.01 - 0.1 mm/mm strain) of the stress-strain curve ($F_{5.167}$, df_{11}), **B:** compressive strength of hydrogels at failure ($F_{0.850}$, df_{11}), **C:** compressive strain of hydrogels at failure ($F_{3.009}$, df_{11}). All measurements were performed at 25 °C. Error bars represent standard deviation, "*" represents a p-value of ≤0.05, "ns" represents nonsignificant; df represents degrees of freedom; one-way ANOVA, followed by Tukey's test, n = 3 independent samples.

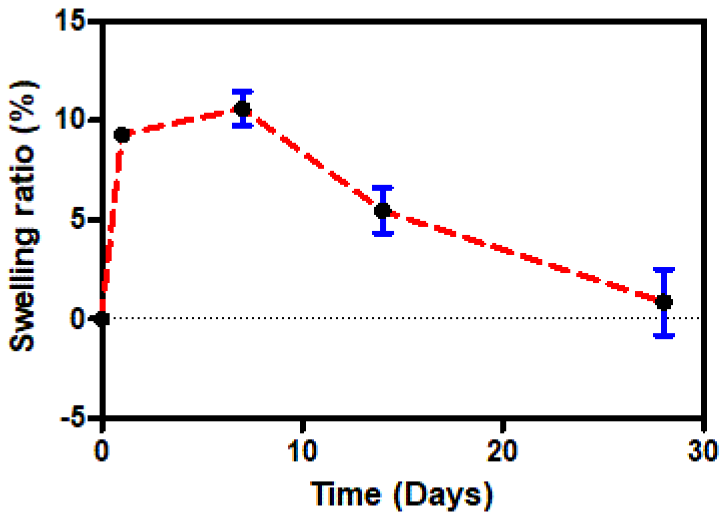

Figure S6. Swelling of collagen furan hydrogels when stored in PBS at 25 °C for 28 days. Formulation used **r2**.

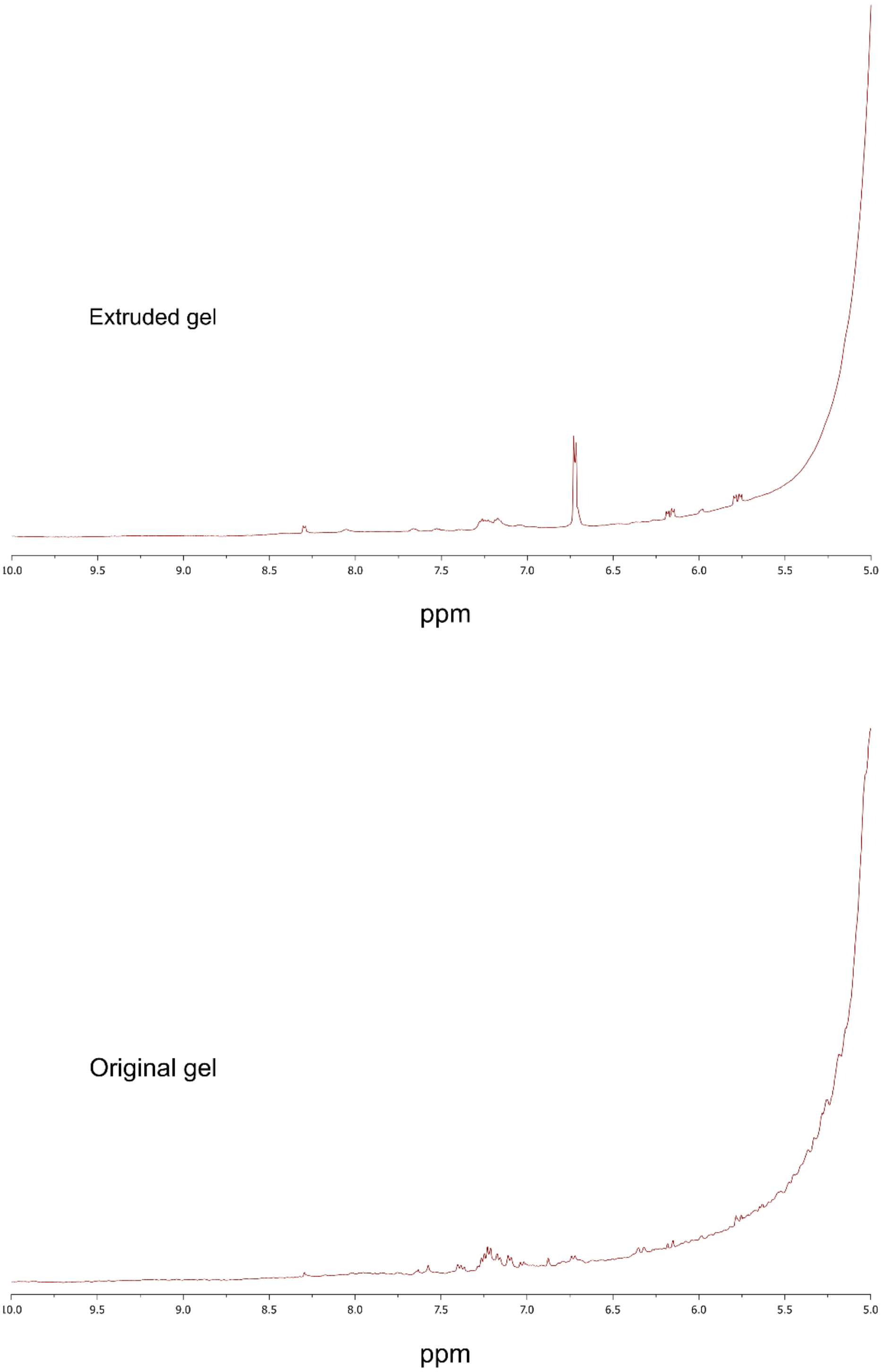

Figure S7. ^{1}H NMR spectra of original and extruded collagen-furan hydrogel after collagenase degradation.

Day 5
100x

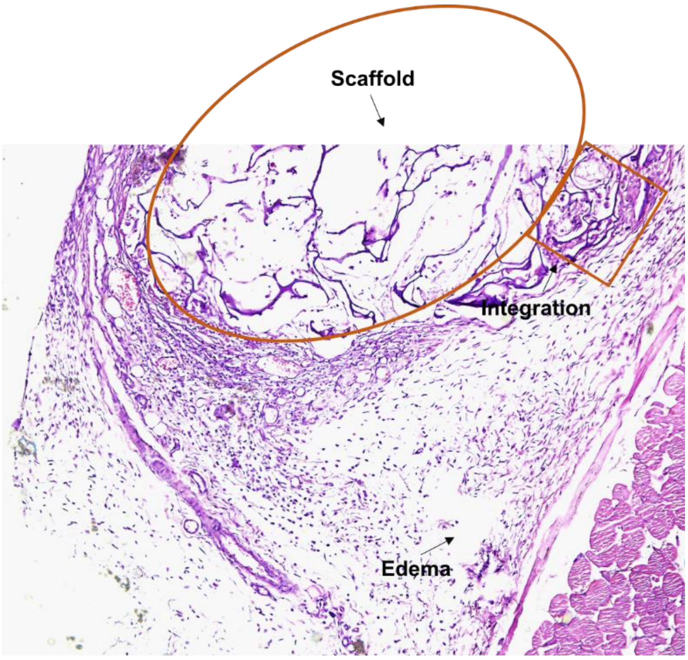

Figure S8. Hematoxylin and eosin (H&E) staining of collagen furan hydrogel (**r2**) with the surrounding tissue after 5 days of implantation, showing the integration and ingrowth of noninflammatory tissue into the scaffold.

Table S1. Different collagen furan hydrogel formulations used in this study.

Name	Formulation<br>Furan to maleimide	Collagen concentration		PEG concentration		Furan concentration	Maleimide concentration
		[%]	[μM]	[%]	[μM]	[μM]	[μM]
**r1**	1:1	2	67	0.58	580	4659	4640
**r2**	1:2	2	67	1.16	1160	4659	9280
**r3**	1:3	2	67	1.74	1740	4659	13920
**r4**	1:4	2	67	2.32	2320	4659	18560
